# The GC-TOF/MS-based Metabolomic analysis reveals altered metabolic profiles in nitrogen-deficient leaves and roots of tea plants (*Camellia sinensis*)

**DOI:** 10.1186/s12870-021-03285-y

**Published:** 2021-11-02

**Authors:** Zheng-He Lin, Chang-Song Chen, Qiu-Sheng Zhong, Qi-Chun Ruan, Zhi-Hui Chen, Xiao-Mei You, Rui-Yang Shan, Xin-Lei Li

**Affiliations:** Tea Research Institute, Fujian Academy of Agricultural Sciences, Fu’an, 355000 China

**Keywords:** Nitrogen deficiency, *Camellia sinensis*, Metabolomics, Tea quality

## Abstract

**Background:**

Nitrogen (N) fertilizer is commonly considered as one of the most important limiting factors in the agricultural production. As a result, a large amount of N fertilizer is used to improve the yield in modern tea production. Unfortunately, the large amount of N fertilizer input has led to increased plant nitrogen-tolerance and decreased amplitude of yield improvement, which results in significant N loss, energy waste and environment pollution. However, the effects of N-deficiency on the metabolic profiles of tea leaves and roots are not well understood.

**Results:**

In this study, seedlings of *Camellia sinensis* (L.) O. Kuntze Chunlv 2 were treated with 3 mM NH_4_NO_3_ (Control) or without NH_4_NO_3_ (N-deficiency) for 4 months by sandy culture. The results suggested that N-deficiency induced tea leaf chlorosis, impaired biomass accumulation, decreased the leaf chlorophyll content and N absorption when they were compared to the Control samples. The untargeted metabolomics based on GC-TOF/MS approach revealed a discrimination of the metabolic profiles between N-deficient tea leaves and roots. The identification and classification of the altered metabolites indicated that N deficiency upregulated the relative abundances of most phenylpropanoids and organic acids, while downregulated the relative abundances of most amino acids in tea leaves. Differentially, N-deficiency induced the accumulation of most carbohydrates, organic acids and amino acids in tea roots. The potential biomarkers screened in N-deficient leaves compared to Control implied that N deficiency might reduce the tea quality. Unlike the N-deficient leaves, the potential biomarkers in N-deficient roots indicated an improved stress response might occur in tea roots.

**Conclusions:**

The results demonstrated N deficiency had different effects on the primary and secondary metabolism in tea leaves and roots. The findings of this study will facilitate a comprehensive understanding of the N-deficient tea plants and provide a valuable reference for the optimized N nutrient management and the sustainable development in the tea plantations.

**Supplementary Information:**

The online version contains supplementary material available at 10.1186/s12870-021-03285-y.

## Background

Nitrogen (N) nutrient is pivotal for the growth of tea plants (*Camellia sinensis* L.). As an essential macronutrient, the input of N fertilizer in the tea plantations dramatically affects the yield of tea leaves [1]. For another, N is indispensable for the biosynthesis of amino acids. The quality of tea leaves, such as mellowness and freshness, are determined by an appropriate level of free amino acids [2]. Apart from the amino acids, the biosynthesis of the quality-related chemical components of tea, such catechin, caffeine and aroma compounds, also require a balance of C/N in tea leaves [3]. Accordingly, the nutrient status of N significantly influences the yield and quality of tea leaves [4].

Much attention has been drawn to the over-application of N fertilizer in tea plantations [5–7]. However, the N-deficiency of tea plantations is not to be ignored. A lower utilization efficiency of N fertilizer, including N leaching and volatilization, led to N-deficiency of tea plants [8]. For instance, it was estimated that over 50% of active N in the N fertilizer lost in the crop fertilization [9]. Chen et al. [10] reported that only less than 25% N fertilizer can be assimilated by the tea plants. Besides, as a perennial leaf-harvest crop, the continuous plucking of the most N-concentrated leaves removes N nutrients from the plants [11]. The leaf N-deficiency is therefore easily found, especially in the mountainous tea plantations of south China with less abundant and high loss of N nutrients [12, 13].

As an ammonium (NH_4_^+^) preferential crop [14, 15], the tea plants respond to the nutrient status of N promptly [16]. For instance, it was evidenced that the biochemical processes of NH_4_^+^ absorption, assimilation and transportation could be observed within 2 h in the tea plants [17]. Considering the alteration of metabolic pathways induced by N-depletion, which would affect the quality of tea leaves, the studies on the altered carbon and nitrogen metabolisms of tea shoots under N-shortage were frequently reported. For example, a decreased amino acid content and an increased flavonoids concentration were found in tea shoot under N-deficiency within 7 days’ duration [18]. Li et al. [19] investigated the carbon and N metabolisms in different tea leaf positions, and found leaves from lower part had less N content, coincident with less caffeine and total amino acids contents. Similarly, it was also revealed that N-shortage decreased the theanine content compared to N-plus leaves of Longjing [20]. Studies mentioned above mainly focused on the metabolic profiles of N-deficient tea shoots. However, the metabolic alterations of N-deficient tea roots were less discussed. Tea roots respond to N-deficiency fast and make a crucial contribution to the synthesis of most amino acids. The comparison of the metabolic profiles on the leaves and roots will increase our understanding of N-deficient tea plants.

Previous studies demonstrated N-deprivation significantly decreased the CO_2_ assimilation of plants including tea and citrus [21, 22]. In the present study, the global metabolic profiles of N-deficient leaves and roots based on an untargeted metabolomics approach were discussed. With a particular focus on the amino acids, organic acids and carbohydrates that related to the tea quality, the study was aimed to provide valuable references for a comprehensive insight into the metabolic regulation of N-deficient tea plants and, ultimately, for better N nutrient management in tea plantations.

## Results

### Effect of N-deficiency on the growth of tea plants

**Fig. 1:**
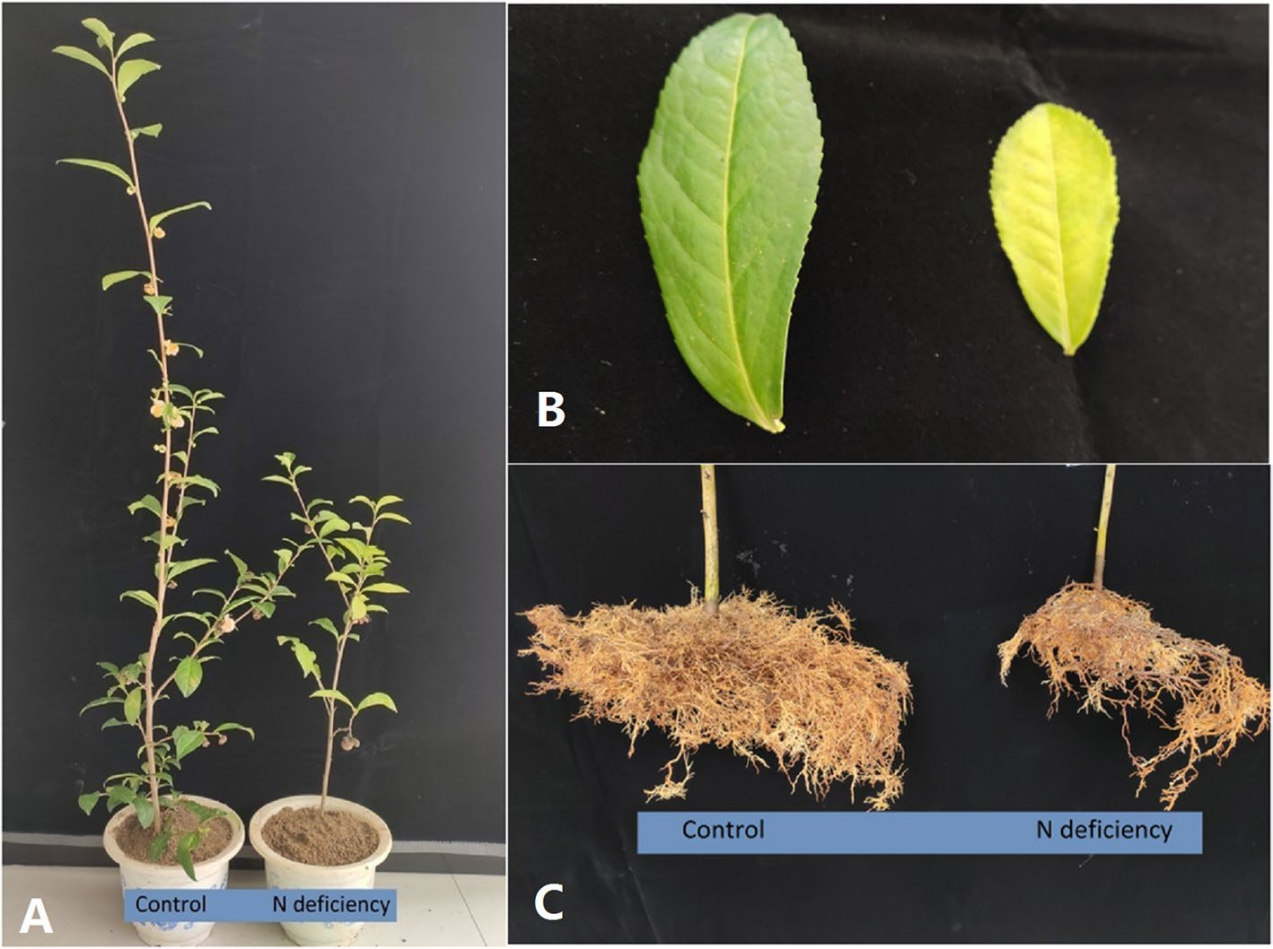
The effects of N-deficiency on the growth (A), leaf (B) and root (C) development of tea trees

An obvious difference was found in the phenotype of the N-deficient tea plant compared to control. As found in Fig. 1A, the tea plant height was significantly decreased by the N-deficiency with a shortening of the internodes. Typically, the leaf chlorosis, developing from lower to upper shoots, was observed in N-deficient treatment compared to control. Overall, the surface area of the chlorotic leaves is smaller than control leaves (Fig. 1B). N-deficiency induced root browning (Fig. 1C), and both primary roots and lateral roots were darker, indicating that N-deficient roots was less vigorous than the control ones.

### Biomass allocation, N content and leaf chlorophyll (Chl) content of tea plants under N-deficiency

**Fig. 2:**
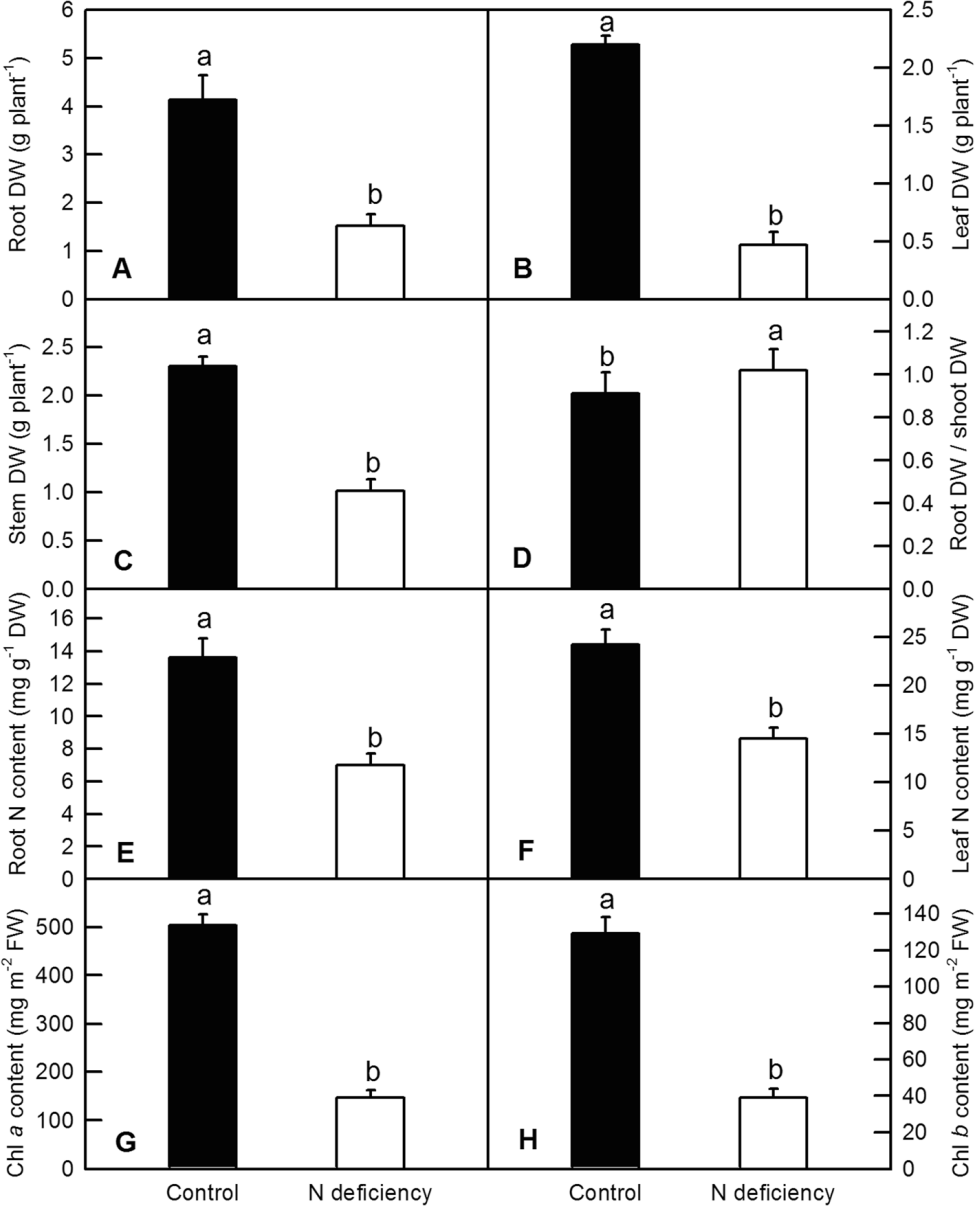
The biomass allocation, N content and leaf Chl content of tea plants under N-deficiency. **A**: root dry weight (DW); **B**: stem DW; **C**: leaf DW; **D**: ratio of root DW/shoot DW; **E**: N content in the roots; **F**: N content in the leaves; **G**: leaf Chl *a* content; **H**: leaf Chl *b* content. Values represent mean ± standard error (SE, *n* = 6). Significant difference (*P* < 0.05) was indicated by different letters above the bars

**Fig. 3:**
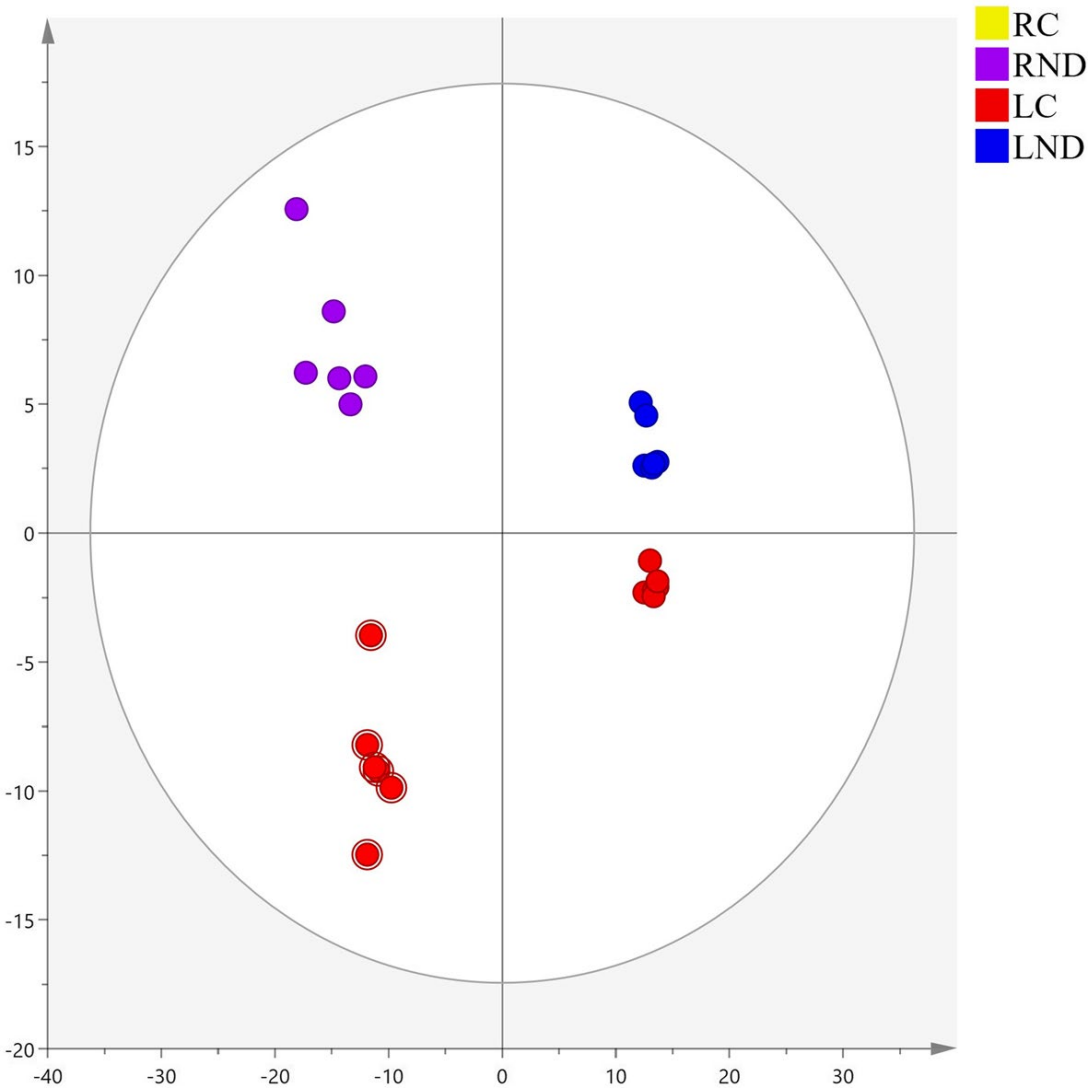
The PCA score scatter plot in N-deficient leaves and roots of tea plants (R2X = 0.617, Q2X = 0.464). LC: control leaves; LND: N-deficient leaves; RC: control roots; RND: N-deficient roots. PCA: principal component analysis. Six biological replicates are performed

The N-starvation remarkably decreased the dry weight (DW) of roots (Fig. 2A), leave (Fig. 2B) and stems (Fig. 2C) of tea plants. By contrast, the ratio of root DW to shoot DW was significantly upregulated by N-deficiency (Fig. 2D). The N content was significantly decreased in N-deficient tea plants. As found in Fig. 3, N-deficient roots and leaves had only 52.17% (Fig. 2E) and 59.65% (Fig. 2F) of N content compared to that of control, respectively. The N-deficient tea plants had obviously lower Chl content compared to control. The Chl *a* (Fig. 2G) and Chl *b* (Fig. 2H) were significantly downregulated in N-deficient leaves of tea plants.

### Metabolic profiles in the N-deficient leaves and roots of tea plants

**Fig. 4:**
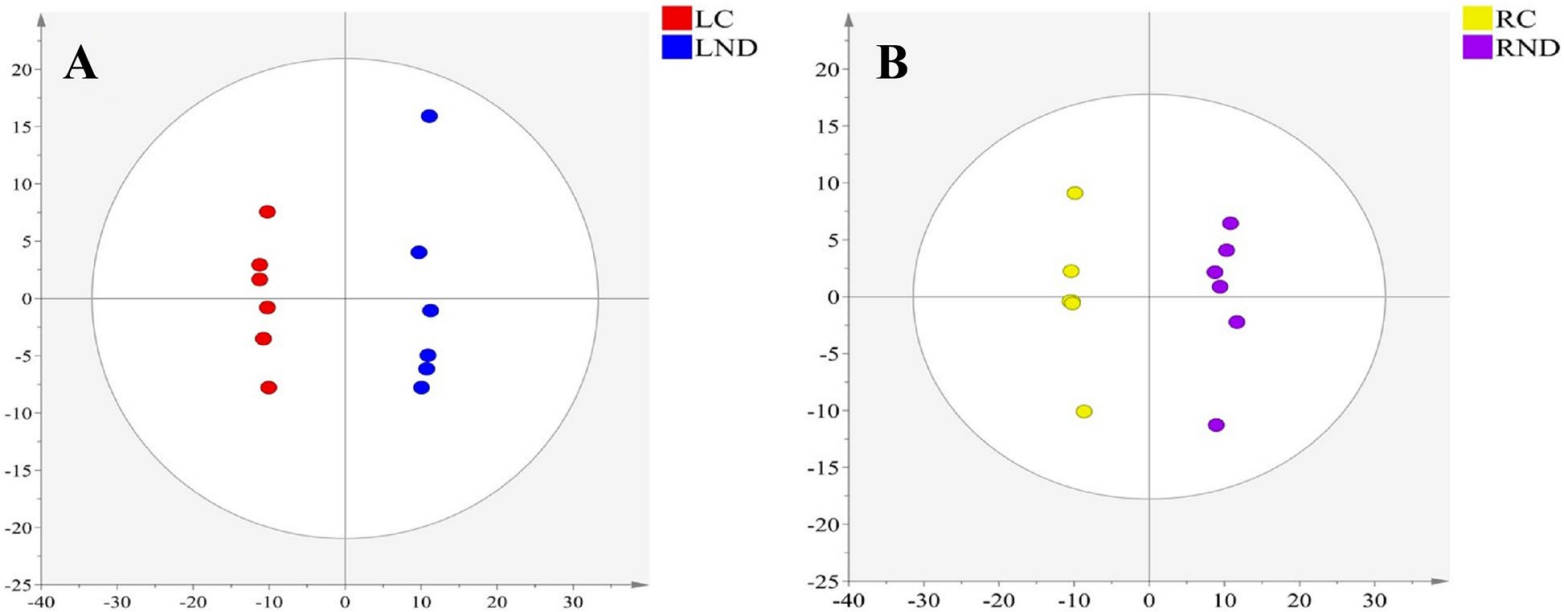
The OPLS-DA score scatter plot in N-deficient leaves (**A**, R2X = 0.442, R2Y = 0.998, Q2 = 0.937) and roots (**B**, R2X = 0.413, R2Y = 0.993, Q2 = 0.889) of tea plants. LC: control leaves; LND: N-deficient leaves; RC: control roots; RND: N-deficient roots. OPLS-DA: orthogonal partial least squares-discriminant analysis. Six biological replicates are performed

The automated deconvolution of mass spectral data produced 422 putatively identified metabolites with a high degree of similarity (≥70%) in both leaves and roots of tea plants (Table S1). After normalization, the mass spectral data were imported into Simca 14.1 for PCA and OPLS-DA analysis. PCA, as an unsupervised analysis method, was performed to investigate the metabolic distinction between groups. As shown in Figs. 3, 24 samples were group into four independent clusters by PCA, indicating a clear separation between control and N-deficient leaves or roots, respectively. The score plot of PCA had three components, representing 61.7% of total variances. The OPLS-DA was performed to create a model prediction and to discriminate the variation between control and N-deficient samples. As found in Fig. 4, a good discrimination was observed between control and N-deficient leaves (Fig. 4A) or roots (Fig. 4B). The score plot accounted for 44.2 and 41.3% of total variance with a prediction parameter Q^2^ = 0.937 and 0.889 in the leaves and roots, respectively. The discrimination indicated a significant alteration of metabolites in leaves and roots under N-deficiency.

### Overview of altered metabolites in the N-deficient leaves and roots of tea plants

The VIP value, calculated in the OPLS-DA, was used to evaluate the explanatory capacity and influential intensity of metabolites and to reflect the importance of metabolites in the model. In the present study, the threshold of significantly altered metabolites by N-deficiency was set when VIP > 1.0. Also, the *p*-value, calculated from Student’s *t*-test, was set at a threshold value of less than 0.05 to indicate the statistical difference between groups. The thresholds of fold change was set at ≥1.20 or ≤ 0.80 for upregulated and downregulated metabolites, respectively.

**Fig. 5:**
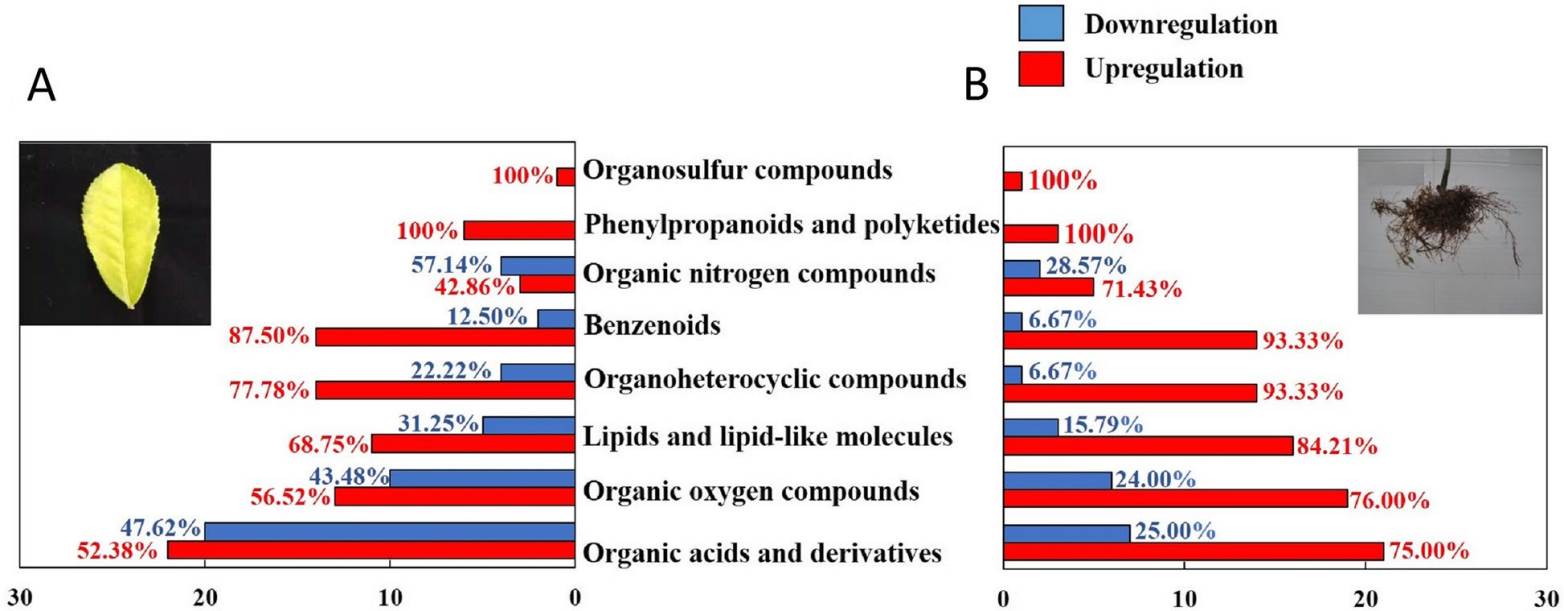
The number of overall altered metabolites in N-deficient leaves (**A**) and roots (**B**) of tea trees. The upregulation of metabolites represents fold change of abundance ≥1.20, *p*-value < 0.05 and VIP > 1.0. The downregulation of metabolites represents fold change of abundance ≤0.80, *p*-value < 0.05 and VIP > 1.0. The percentage near the columns represents the ratio of up-regulated (in red) or down-regulated (in blue) metabolites in each group. The metabolites were classified according to http://classyfire.wishartlab.com/

The altered metabolites were screened on VIP value, *p*-value and fold change. Accordingly, 140 and 125 metabolites were identified as significantly altered metabolites in the N-deficient leaves and roots of tea plants compared to control, respectively (Table S2;Table S3). Most of those altered metabolites were classified into eight groups containing organic acids and derivates, organic oxygen compounds, lipid and lipid-like molecules, organoheterocyclic compounds, benzenoids, organic nitrogen compounds, phenylpropanoids and polyketides and organosulfur compounds (Fig. 5). In each metabolites’ group, over half of the benzenoids, organoheterocyclic compounds, lipids and lipid-like molecules, organic acids and derivates and organic oxygen compounds were upregulated by N-deficiency in tea leaves. Notably, the corresponding ratios of upregulated metabolites were higher in the N-deficient roots, suggesting more obvious alteration on the metabolome of N-deficient roots compared to that of leaves.

### Altered metabolic pathways in the N-deficient leaves and roots of tea plants

**Fig. 6:**
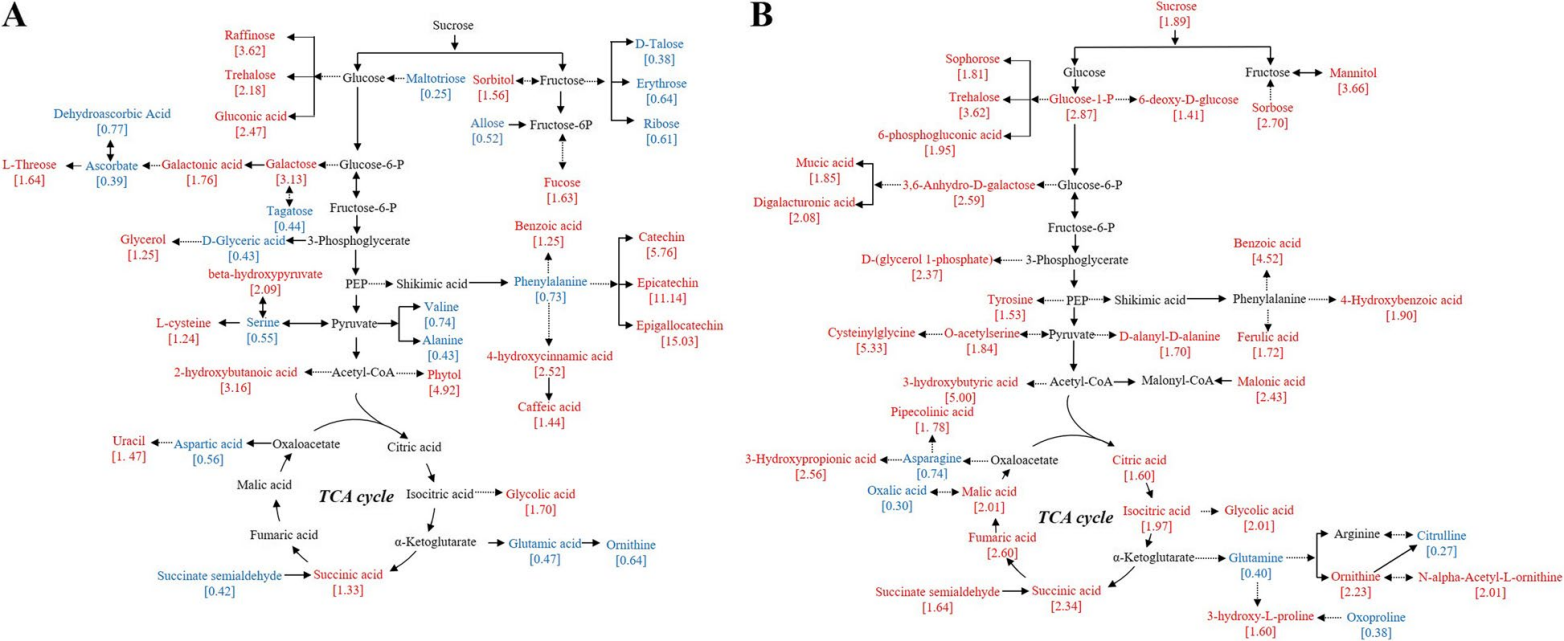
Metabolic pathways in N-deficient leaves (**A**) and roots (**B**) of tea plants. The number in the square bracket under the metabolites represent fold change of N-deficient leaves or roots compared to control of tea plants. The upregulated metabolites were labeled by red color (Fold change> 1.2, *p*-value < 0.05 and VIP > 1.0). The downregulated metabolites were labeled by blue color (Fold change< 0.8, *p*-value < 0.05 and VIP > 1.0). The pathways were designed according to KEGG pathways (http:www.kegg.jp/kegg/pathway.html.com). Six biological replicates are performed

The primary metabolic pathways, mainly regarding to tricarboxylic acid (TCA) cycle, glycolysis and pentose phosphate pathway (PPP), were enriched (Fig. 6). As shown in Fig. 6A, the relative abundances of metabolites in the galactose metabolism (map00052) and starch & sucrose metabolism (map00500) such as raffinose (fold change: 3.62), galactose (3.13), and trehalose (2.18), were upregulated in N-deficient tea leaves. Similarly, the relative abundances of sorbitol (1.56) and fucose (1.63) in the fructose and mannose metabolism (map00051) were upregulated. Differentially, the relative abundances of monosaccharides such as D-talose (0.38), erythrose (0.64), and ribose (0.61) in the pentose phosphate pathway (map00030) were downregulated. The N-shortage downregulated the relative abundance of phenylalanine (0.73). By contrast, the relative abundance of benzoic acid (1.25), associated with the phenylalanine metabolism (map00360) was upregulated (Table S2). Additionally, relative abundances of caffeic acid in the phenylpropanoid biosynthesis (map00940), catechin (5.76), epicatechin (11.14) and epigallocatechin (15.03) in the flavonoid biosynthesis (map00941) and were also promoted. Beside of phenylalanine, the relative abundances of other amino acids such as valine (0.74), alanine (0.43), serine (0.55), aspartic acid (0.56), glutamic acid (0.47), ornithine (0.64), except for L-cysteine (1.24), were downregulated by N-starvation in tea leaves (Table S2). N-deficiency upregulated the relative abundances of carboxylic acids, such as succinic acid (1.33) in the TCA cycle (map00020). Furthermore, relative abundance of hydroxy acids, such as 2-hydroxybutanoic acid (3.16) in the propanoate metabolism (map00640), beta-hydroxypyruvate (2.09) in the glycine, serine and threonine metabolism (ec00260), and glycolic acid (1.74) in the glyoxylate and dicarboxylate metabolism (map00630) were also upregulated in N-deficient tea leaves (Table S2).

In the tea roots, N-deficiency promoted the relative abundances of sucrose [1.89], glucose-1-P (2.87), sorphorose [1.81], trehalose (3.62), 6-phosohogluconic acid (1.95) and 3,6-anhydro-D-galactose (2.59). Additionally, the relative abundances benzoic acid (4.52), ferulic acid (1.72) and 4-hydroxybenzoic acid (1.90), synthesized from the phenylalanine, were upregulated in N-deficient roots. Moreover, N-deficiency enhanced the TCA cycle (map00020) in the tea roots, which was supported by upregulation of citric acid (1.60), isocitric acid (1.97), succinic acid (2.34), fumaric acid (2.60) and malic acid (2.01). Different from the N-deficient tea leaves, the N-deficient tea roots had an accumulation of most amino acids, such as D-alanyl-D-alanine (1.69), gly-pro (4.58), ornithine (2.23), 3-(1-Pyrazolyl)-L-alanine (1.78), 3-hydroxy-L-proline (1.60), O-acetylserine (1.84), cysteinylglycine (5.33), N-alpha-Acetyl-L-ornithine (2.01) and tyrosine (1.53). Overall, it was found that only six out of 18 altered amino acids, peptides and analogues were downregulated in N-deficient tea roots (Table S3).

### Change of organic acids, amino acids and carbohydrates in N-deficient leaves and roots of tea plants

**Fig. 7:**
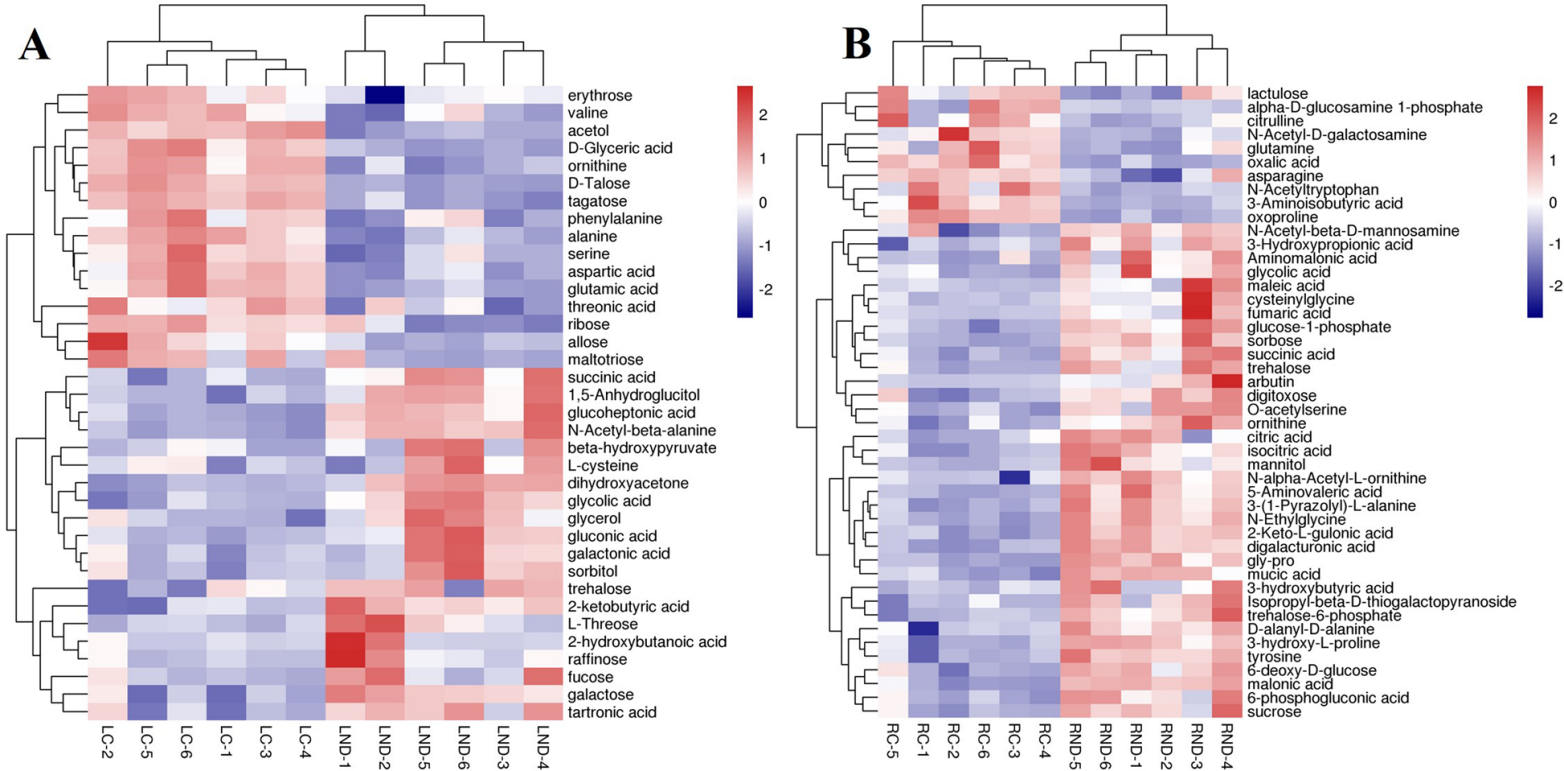
The heatmaps of most organic acids and derivatives, organic oxygen compounds in N-deficient leaves (**A**) and roots (**B**) of tea plants. LC: control leaves; LND: N-deficient leaves; RC: control roots; RND: N-deficient roots. The color from navy, white and firebrick represent the relative abundance of metabolites increased. The heatmap was performed at https://www.omicshare.com/tools/. Six biological replicates are performed

The abundances of organic acids, amino acids as well as carbohydrates were summarized and presented in the heatmap (Fig. 7). N-deficiency downregulated the abundances of most amino acids including valine (0.74), ornithine (0.64), phenylalanine (0.74), alanine (0.43), serine (0.55), aspartic acid (0.56) and glutamic acid (0.47) while upregulated most of organic acids such as succinic acid (1.33), glucoheptonic acid (1.15), beta-hydroxypyruvate (2.09), glycolic acid (1.70), gluconic acid (2.47), galactonic acid (1.76), 2-ketobutyric acid (2.22), 2-hydroxybutanoic acid (3.16) and tartronic acid (1.26) in the leaves (Fig. 7A). N-deficiency downregulated the relative abundances of several carbohydrates such as erythrose (0.64), tagatose (0.44), ribose (0.61), allose (0.52), talose (0.38), maltotriose (0.25) while upregulated the abundances of trehalose (2.18), fucose (1.63), galactose (3.13), L-threose (1.64), raffinose (3.62) in the leaves (Table S2).

In tea roots, N-deficiency upregulated the abundances of most carbohydrates, such as sucrose (1.89), glucose-1-phosphate (2.87), sorbose (2.70), trehalose (4.10) and so on. Likewise, the relative abundances of most organic acids and amino acids were also enhanced under N-shortage, such as citric acid (1.60), maleic acid (2.01), succinic acid (2.34) and so on (Fig. 7B;Table S3).

### The potential biomarkers for N-deficiency in tea leaves and roots

**Fig. 8:**
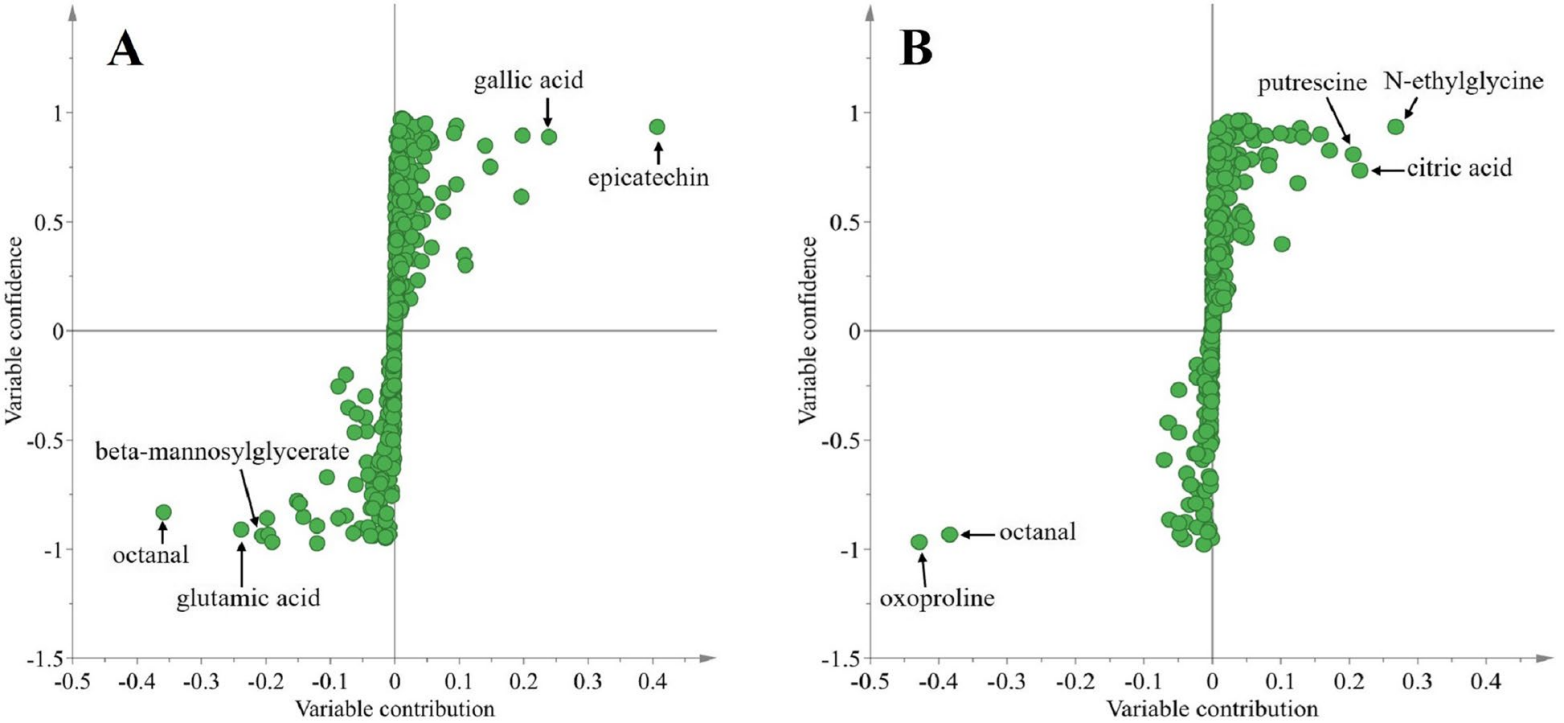
Scatter plots from OPLS-DA of N-deficient leaves (**A**) and roots (**B**) of tea plants. The potential biomarkers that induced by N-deficiency were nominated according to the variable contribution > 0.2 or < − 0.2

The variable contributions of metabolites were analyzed according to the OPLS-DA model to screen the variables that responsible for the discrimination between Control and N-deficient leaves and roots of tea plants. The metabolites with a variable contribution > 0.2 or < − 0.2 were considered as potential biomarkers to clustering the groups of control and N-deficiency. As found in Fig. 8A, five potential biomarkers were found in N-deficient tea leaves, including epicatechin (11.14), gallic acid (1.42), octanal (0.08), glutamic acid (0.47) and beta-mannosylglycerate (0.55) (Table S2). Similarly, five potential biomarkers were also obtained in tea roots, including N-ethylglycine (1.80), citric acid (1.60), putrescine (1.62), oxoproline (0.38) and octanal (0.08) (Fig. 8B; Table S3).

## Discussion

### Physiological responses of tea plants to N deficiency

The N nutrient, especially the ammonium, plays multiple roles in the shoot development and quality formation of tea plants [23, 24]. The understanding of tea plant’s responses to N-deficiency provides essential information for an optimized N replenishment in the tea plantations, which would contribute to a higher yield and better quality of tea (Fig. 1). The physiological responses such as a significant decline of biomass in the roots (Fig. 2A), shoots (Fig. 2B and C) and Chl content (Fig. 2G and H) in N-deficient tea plants were in line with the results reported by Fan et al .[25]. N is a mobile nutrient element in plants, meaning its translocation from the source roots to the sink leaves under N-deficiency. Consequently, the leaves accumulated higher N content than the roots under N-deficiency (Fig. 2E and F).

### Overall metabolic profiles of N-deficient leaves and roots in tea plants

The metabolic profiles of N-deficient tea leaves and roots were investigated based on untargeted metabolomics approach. A clear separation of four clusters, containing Control/N-deficient leaves and roots in the PCA, indicated a significant difference in the metabolic composition among groups (Fig. 3). A similar clustering was observed in the N-deficient *Isatis indigotica*, a Chinese traditional herb [26]. A clearer separation of control and N-deficient leaves (Fig. 4A) or roots (Fig. 4B) of tea plants was found by the OPLS-DA, suggesting more apparent metabolic alteration induced by N deficiency in leaves and roots of tea plants, respectively. A similar finding was also reported in N-deficient leaves and roots of tomato plants [27].

### Effects of N deficiency on the quality-related components of tea leaves

Strikingly, tea quality components, including epicatechin (11.14), (+)-catechin (5.76), epigallocatechin (15.03) and caffeic acid (1.44) were upregulated in the N-deficient tea leaves compared to Control (Fig. 5;Table S2). The increased abundance of catechin in N-deficient tea leaves is consistent with the results reported by Fan et al. [25]. It was found that the optimal level of catechin generated the proper astringency in the roasted green tea [28]. Therefore, the increased catechin might impair the tea flavor by promoting the astringency of tea leaf. The significant accumulation of catechin and epicatechin in low N-treated tea was also reported by Ruan et al. [29]. Zhou et al. [30] demonstrated that the upregulation of genes encoding flavonoid 3′-hydroxylase is responsible for the accumulation of catechin in N-depleted tea leaves. Besides, most of upregulated benzenoids in the N-deficient leaves were phenols, benzene and substituted derivatives. The upregulation of most benzoic acids and phenolic compounds were also reported in N-deficient *Matricaria chamomilla* leaf rosettes, considering as a preparation for phenylpropanoid metabolism [31].

On the other hand, the “Umami” taste of tea leaves was determined by the level of theanine. The de novo biosynthesis of theanine dependent on the levels of glutamic acid and ethylamine [32–34]. The alanine is a precursor of ethylamine. The downregulated contents of alanine (0.43) and glutamic acid (0.47) in the N-deficient leaves indicated a less content of theanine in the N-deficient tea leaves compared to Control (Table S2). N-deficiency decreased the theanine content in the tea shoots has been demonstrated by Liu et al. [20]. Besides, the theanine was mainly synthesized in the roots then transported to the shoots by related transporters [35]. Under N deficiency, the theanine transporters mediated the transfer of amino acids including theanine from roots to new shoots [17]. The present results also indicated a potentially impaired transportation of amino acids from roots to shoots, which was supported by the downregulation of most amino acids in the N-deficient leaves (Fig. 7A), while upregulation of most amino acids in the N-deficient roots (Fig. 7B). Similar results about downregulation of most amino acids under N-shortage were also found in leaves of bell pepper [36] and rice [37].

### Effects of N deficiency on the abundance of organic acids in leaves and roots of tea plants

Organic acids are generally considered as the intermediate products of respiration and photosynthesis in higher plants and are essential for ammonia assimilation and amino acid synthesis [38–40]. The results that N-deficiency increased malic acid and citric acid in the roots rather than in the leaves is consistent with our previous study in phosphorous-deficient tea plants (Table S2) [41]. N-deficiency enhanced the abundances of organic acids in the TCA cycle of tea roots, meaning that an increasing carbon precursors for the amino acid biosynthesis might contribute to the upregulation of most amino acids in the N-deficient tea roots (Fig. 6B; Table S3) [42]. A similar enhancement of the TCA cycle was also reported in the low-N tolerant soybean seedlings [43]. Besides, the accumulation of organic acids was crucial for plants to cope with the nutrient stress by improving nutrient absorption efficiency in the rhizosphere of tea plants [41].

### Effects of N deficiency on the abundance of carbohydrates in leaves and roots of tea plants

The carbohydrate metabolism, affected by many crucial physiological processes, such as photosynthesis and respiration, is associated with the adaptability to stress in plants [44]. Our results indicated a much noticeable increment of carbohydrate and carbohydrate conjugates, belonging to the superclass of organic oxygen compounds, in the N-deficient roots compared to the leaves (Fig. 5). The carbohydrates in the roots might be transported from the leaves to support the root growth under N-deficiency condition. Ruan et al. [45] reported the sucrose was an energy substance for root development and nutrient uptake. Therefore, the translocation of sucrose from N-deficient leaves to roots would balance the root demand for respiration and shoot demand for N absorption. The increased allocation of carbohydrates from leaves to root under N-deficiency was also reported in spinach [46]. Besides, the study on microalgae indicated both carbohydrates and lipid increased under the N-deficiency [47].

However, the accumulation of carbohydrates and lipid in the N-deficient tea roots increased the osmotic pressure, revealed by increased mannitol (3.66), an indicator of osmotic stress, which would result in over-accumulation of reactive oxygen species (ROS) (Table S3). Accordingly, molecules of osmotic regulators were accumulated. For instance, the trehalose was upregulated in N-deficient tea roots, protecting the cell membrane and protein from damage [48]. Sorbose, an abiotic and abiotic stress-responsive metabolite, was also upregulated in the N-deficient tea roots [49]. In N-deficient tea leaves, some monosaccharides were upregulated, such as galactose [3.13], trehalose (2.18) and fucose (1.63) (Table S2). Adversely, erythrose (0.64), ribose (0.61), allose (0.52) and tagatose (0.44) were downregulated by N-deficiency. Therefore, the alteration of carbohydrates in the N-deficient leaves did not show an obvious change (Table S2).

### Potential biomarkers of N deficiency in tea leaves and roots

The potential biomarkers or N-deficient leaves and roots were examined by the variable contribution of OPLS-DA (Fig. 8). Most of those potential biomarkers found in the N-deficient leaves are associated with the tea quality. For instance, the octanal was reported as a critical odorant volatile that generates the chestnut-like aroma of tea [50]. The glutamic acid is essential for theanine synthesis [51].

The beta-mannosylglycerate is a potential protein thermostabilizer [52]. A downregulation of beta-mannosylglycerate (0.55) indicated that N deficiency decreased the leave protein stability. Differentially, N deficiency upregulated the relative abundances of epicatechin (11.14) and gallic (1.42) in the leaves. In the tea leaves, epicatechin is a derivative of catechin, whose abundance was negatively correlated with the tea sensory evaluation [53, 54] (Table S2). Gallic acid is one of the most common moieties in the structure of tea phenolics [55], whose biosynthesis was relied on phenylpropanoid pathway or the dehydrogenation of shikimic acid pathway [56]. The downregulation of octanal (0.08) and glutamic acid (0.47), whereas the upregulation of catechin (11.14) supported that the flavor tea leaves was negatively affected by the N depletion (Table S2).

Differentially, most of the potential biomarkers in the N-deficient roots were associated with the stress response. For instance, the N-deficiency downregulated the relative abundance of oxoproline (0.38), a precursor of proline [57], implied its increased conversion to proline, promoting the osmotic regulation in the N-deficient tea roots. By contrast, the putrescine (1.61), N-ethylglycine (1.80) and citric acid (1.60) were upregulated by N deficiency (Table S3). Previous study indicated that putrescine, a polyamine, played an important role in improving photosynthesis and antioxidant enzyme activity of tea plants under stress [58]. Citric acid was also reported to be associated with antioxidant activity by increasing the polyphenols content. Likely, the oxidation of N-ethylglycine generated the glyoxylate, ethylamine and hydrogen peroxide. The production of hydrogen peroxide strengthens the reactive oxygen species scavenging in plants [59].

## Conclusions

In this study, the physiological responses and metabolic profiles of N-deficient tea leaves and roots were characterized. The results suggested that N-deficiency impaired the seedling growth of tea plants, decreased the N absorption and the leaf Chl content significantly, leading to leaf chlorosis. Based on the untargeted metabolomics approach, the study demonstrated that N-deficiency induced metabolic alteration in both leaves and roots of tea plants. Strikingly, N deficiency upregulated the relative abundances of most phenylpropanoids, organic acids, whereas downregulated the relative abundances of most amino acids in the tea leaves. Differentially, N-deficiency induced the accumulation of most carbohydrates, organic acids and amino acids in tea roots. The potential biomarkers screened in the leaves reflected a tea quality, while the potential biomarkers in the N-deficient roots were mainly associated with the improved stress response. Those results contributed to a better understanding of N-deficiency in tea plants, which would provide references for N nutrient management in the tea plantations.

## Materials and methods

### Plant culture and N treatments

Tea seedlings were raised from cuttings of cultivar “Chunlv 2” [*Camellia sinensis* (L.) O. Kuntze], which is bred from tea cultivar “Fuyun 6” by Tea Research Institute, Fujian Academy of Agricultural Sciences (Fu’an, China). “Chunlv 2” was newly bred tea cultivar by the authors listed in this study. Ten-months-old own-rooted tea seedlings were cultivated in 6 L pots containing clean river sands in a greenhouse with natural photoperiod at Tea Research Institute, Fujian Academy of Agricultural Sciences (Fu’an, China) from Feb 2018 to Jun 2018. After 7 days of transplanting, the seedlings were irrigated with half-strength nutrient solution for 14 days. Then, the seedlings were irrigated until dripping (500 ml) every 2 days with full-strength nutrient solution [0.5 mM Ca(H_2_PO_4_)_2_, 3 mM NH_4_NO_3_, 0.5 mM CaCl_2_, 1.0 mM K_2_SO_4_, 46 μM H_3_BO_3_, 0.6 mM MgSO_4_, 9 μM MnSO_4_, 2 μM CuSO_4_, 9 μM ZnSO_4_, 2.6 μM Na_2_MoO_4_ and 30 μM Fe-EDTA] with nitrogen concentration of 0 mM (N-deficiency) or 3 mM NH_4_NO_3_ (Control) for 4 months. The pH of all the nutrient solutions were adjusted to 5.0 to 5.1 [21, 60]. At the end of the experiment, new shoots with one bud and two leaves were punched and root apices (about 5 mm) were collected at noon on a sunny day. Leaf and root samples were frozen by liquid nitrogen and stored at − 80 °C until the determination of metabolic analysis. The experiment had a completely randomized design with the two treatments replicated six times and each replication containing one plant.

### Measurement of dry weight and N content

At the end of the experiment, tea plants were harvested from different pots (one plant per pot). These plants are divided into different parts (roots, stems, and leaves). The plant tissues were then dried at 80 °C for 48 h. Total nitrogen was measured using a continuous flow auto-analyser (AAIII; SEAL Analytical, Germany). There were six replicates per treatment.

### Measurement of chlorophyll content

Leaf chlorophyll (Chl) was extracted with 80% acetone (v/v) and assayed according to the method by Lichtenthaler [61]. Briefly, Chl *a* and Chl *b* were assayed at 663 and 645 nm, respectively, in a 1.0 mL extract. There were six replicates per treatment.

### Gas chromatography-mass spectrometry

About 60 mg samples were accurately weighed and were ground with a pre-cooled mortar and pestle in 360 μL pre-cooled methanol and 40 μL 0.3 mg·mL^− 1^ l-2-chlorine-phenylalanine. The ultrasonic extraction was performed for 30 min after fully grinding. The crude extract was then centrifuged for 10 min at 12000 rpm under 4 °C and the supernatant was transferred into glass derived bottle. Samples were blow-dried by moderate nitrogen; 80 μL of 15 mg·mL^− 1^ methoxyamine pyridine solution was then added, vortexed for 60 s and reacted for 90 min at 37 °C. Finally, 100 μL BSTFA [bis(trimethylsilyl)trifluoroacetamide] reagent, containing 1% TMCS (trimethylchlorosilane), v/v, was added into the mixture, reacted for 60 min at 70 °C. The sample was taken out and left at room temperature for 30 min. After the above reactions, metabolite analysis was performed using an Agilent 7890B-5977Agas chromatograph system coupled with a Pegasus 4D time-of-flight mass spectrometer (GC-TOF/MS; Agilent Technologies Inc. CA, USA). The system utilized a DB-5MS capillary column coated with 5% diphenyl cross-linked with 95% dimethylpolysiloxane (30 m × 0.25 mm × 0.25 μm, Agilent). The carrier gas was high purity helium gas (purity no less than 99.999%) and the gas flow rate was 1.0 mL·min^− 1^. The temperature of the injection port was 260 °C. A 1 μL aliquot of the analyte was injected in splitless mode and the solvent was added after 5 min.

The initial column temperature was kept at 60 °C and raised to 125 °C at a rate of 8 °C·min^− 1^, then raised to 210 °C at a rate of 4 °C·min^− 1^, then raised to 270 °C at a rate of 5 °C ·min^− 1^, finally to 305 °C at a rate of 10 °C·min^− 1^ for 3 min. The injection, four stage bar and ion source temperatures were 280 °C, 150 °C and 230 °C, respectively. The energy was 70 eV in electron impact mode.

The mass spectrometry data were acquired in full-scan mode with the mass-to-charge (m/z) ratio range of 50–500 at a rate of 12.02 spectra per second. The QC samples were injected at regular intervals (six replicates for each treatment) throughout the analytical run to provide a set of data from which repeatability could be assessed.

### Data processing

The raw data (D format) was converted to the common format (CDF format) by ChemStation (Version e.02.02.1431, Agilent) software. All chromatograms were processed by ChromaTOF software (Version 4.34, LECO, St Joseph, MI). The peak list was exported as a CSV file containing sample information, name of each material peak, retention times, raw spectra with absolute intensities. The internal standard was used for data quality control, which could reflect the reproducibility of the analysis. Metabolite annotation with the NIST 05 Standard mass spectral database and Fiehn database linked to the ChromaTOF software were manually checked with a similarity of more than 70%. When the results of the two databases were not consistent, the high degree of similarity of the metabolite name was chosen as the annotation result.

### Data analysis

The normalized data sets were analyzed using SIMCA-P 14.0 (Umetrics, Umea, Sweden) for principal component analysis (PCA) and orthogonal partial least squares-discriminate analysis (OPLS-DA). Significantly altered metabolites were screened with variable importance in the projection (VIP) > 1.0, *P* < 0.05, and fold change > 1.20 (up-regulated) or < 0.80 (down-regulated). The heatmaps were generated at http://www.omicshare.com/tool.

The metabolic pathway analysis was performed based on the Kyoto Encyclopedia of Genes and Genomes (KEGG) database. The scatter plots (S-plot) were performed to screen the potential biomarkers using SIMCA-P 14.0.

## Supplementary Information

**Additional file 1: Table S1**. The mass spectral data of control and N-deficient leaves and roots in tea;

**Additional file 2: Table S2**. The altered metabolites in response to N-deficiency in tea leaves;

**Additional file 3: Table S3**. The altered metabolites in response to N-deficiency in tea roots.

## Data Availability

The raw data of the presented results of this study are available on request to the corresponding author.

## Abbreviations

N: Nitrogen
DW: Dry weight
Chl: Chlorophyll
PCA: Principal component analysis
OPLS-DA: Orthogonal partial least squares discrimination analysis
LC: Control leaves
LND: N-deficient leaves
RC: Control roots
RND: N-deficient roots
TCA: Tricarboxylic acid
PPP: Pentose phosphate pathway
